# A Review of the Benefits 3D Printing Brings to Patients with Neurological Diseases

**DOI:** 10.3390/pharmaceutics15030892

**Published:** 2023-03-09

**Authors:** Christine Gander, Kejing Shi, Ali Nokhodchi, Matthew Lam

**Affiliations:** 1 Pharmaceutics Research Laboratory, Arundel Building, School of Life Sciences, University of Sussex, Brighton BN1 9QJ, UK; 2 Medicines Research Group, School of Health, Sport and Bioscience, University of East London, Water Lane, London E15 4LZ, UK; 3 Lupin Pharmaceutical Research Center, 4006 NW 124th Ave., Coral Springs, FL 33065, USA; 4 Department of Chemical and Pharmaceutical Sciences, School of Human Sciences, London Metropolitan University, 166-220 Holloway Road, London N7 8DB, UK

**Keywords:** 3D drug printing, personalized medicine, neurosurgery, bioprinting, implants

## Abstract

This interdisciplinary review focuses on how flexible three-dimensional printing (3DP) technology can aid patients with neurological diseases. It covers a wide variety of current and possible applications ranging from neurosurgery to customizable polypill along with a brief description of the various 3DP techniques. The article goes into detail about how 3DP technology can aid delicate neurosurgical planning and its consequent outcome for patients. It also covers areas such as how the 3DP model can be utilized in patient counseling along with designing specific implants involved in cranioplasty and customization of a specialized instrument such as 3DP optogenetic probes. Furthermore, the review includes how a 3DP nasal cast can contribute to the development of nose-to-brain drug delivery along with looking into how bioprinting could be used for regenerating nerves and how 3D-printed drugs could offer practical benefits to patients suffering from neurological diseases via polypill.

## 1. A Brief Introduction to Neurological Diseases and 3D Print Technology

Neurological disorders refer to diseases of the central and peripheral nervous systems [1]. The more prevalent disorders include Alzheimer’s disease, stroke, epilepsy, and Parkinson’s disease [2]. Many of them severely lower patients’ health-related quality of life and some of them are life-threatening. The degeneration of the cells in the brain via Alzheimer’s disease alone, which is the main cause of dementia, affects around 50 million people worldwide and is projected to double every 5 years. This debilitating disease causes memory loss and changes in personality, which greatly burdens patients, their families, and the economy with an estimated USD 1 trillion annually [3]. Due to the high morbidity caused by neurological disorders, more attention should be paid to support improvement in care and outcomes for people who have neurological disorders. According to the UK National Health Service (NHS), there are 12.5 million episodes of care every year on average.

Recent advancements in 3D technology such as reduction in cost and more advanced computer engineering have allowed its expansion in many applications [4]. The 3DP method has multiple benefits over traditional manufacturing processes [5,6]. Extremely complex designs can be printed, which is ideal for the geometry of the brain and human nervous systems. Various 3DP technologies have been applied to produce accurate and patient-specific models, such as parent artery, skull, vascular network, brain model and head phantom for surgical planning, simulation and training, tissue-engineered implants, and secondary devices [7]. Outside of neurological diseases, 3DP has a broad current and potential application in the medical field of studies with regenerative medicine, artificial heart pump and 3DP eye cornea as just a few examples [4,8,9]. Beyond the medical field, the 3DP technology application is also very broad, ranging from aviation to food industry [10,11].

The flexibility of design in 3DP is a massive advantage for the medical field as it allows for models to be customized for individual patients. In addition, there is low skill involved in the manufacturing process, meaning anyone can learn how to use this technology, making it applicable in the field of neuroscience [12]. Furthermore, 3DP allows for rapid prototyping and printing on demand, which could be ideal for scientific experiments.

## 2. Process

The 3D printing of objects was first developed in the 1980s, and since then, numerous different devices such as stereolithography, PolyJet, selective laser sintering, fused deposition modeling, and drop-on solid deposition have been produced [4]. Generally, 3DP techniques can be classified into powder solidification, liquid solidification, and extrusion-based system. There are many 3D printers which have been developed based on these three mechanisms. For example, drop-on-drop deposition and stereolithography are based on liquid solidification, whereas drop-on-solid deposition and selective laser sintering are based on powder solidification. Fused deposition modeling is based on the extrusion-based system. The fundamental idea that underlies all processes is that the model develops in layers: a virtual model is broken down into 2D layers which are printed layer by layer, piling up over time to create a 3D object [13]. There are three main stages: pre-printing, the printing process, and post-processes. The pre-printing stage includes computed aided design (CAD), which is software that allows the user to design a model virtually. Before the 3D virtual model is printed, the data are broken up into layers that are compatible with the printing device [13]. Medical images can be reconstructed on this software, making it easier to design specific parts for patients with the correct dimensions [14]. Occasionally, support is programmed to help keep the structural integrity of the model during the printing process. This section will cover five widely used 3DP technologies, which are stereolithography (SLA), PolyJet printing, selective laser sintering (SLS), fused deposition modelling (FDM), and drop-on-solid deposition (DOS).

### 2.1. Stereolithography (SLA)

An SLA 3D printer creates models with high precision with a good surface finish. The machine contains a tank filled with photosensitive liquid resin which solidifies when an ultraviolet (UV) laser is concentrated and directed by a mirror. This mirror is controlled by the program created in CAD and works one layer at a time, starting at the bottom of the model and working up. Between each layer, there is a recoating blade that smooths and cleans each layer for the next. The platform the model is on descends, submerging the layer into the resin, and the laser begins again. Once the model is complete, it is extracted from the tank and placed into a UV compartment where the plastic is cured [15].

### 2.2. PolyJet Printing

PolyJet printing is based on the extrusion of a photopolymer in layers which is then cured directly afterward by UV light, removing the need for post-curing. This allows the fabrication of a complex multi-material object. The geometry is protected by gel supports which are quickly removed by water jetting or by hand after the printing has been completed. PolyJet printing is widely used in fabrication relating to surgical planning [16].

### 2.3. Selective Laser Sintering (SLS)

SLS is an expensive process that creates abrasive and opaque models to a high level of precision. This process uses a CO_2_ laser beam on a powder bed that heats the particles, fusing them together, and making a dense sheet. Once the first layer is sintered, the powder tray moves down and a fresh layer of powder is placed and leveled with a roller. The next layer is sintered and the process is repeated until the model is finished. The leftover powder can be recycled and the model is later sandblasted [17].

### 2.4. Fused Deposition Modeling (FDM)

FDM uses a thermoplastic filament which is heated and extruded through a fine nozzle. The nozzle is moved on an X and Y axis based on the model program. The nozzle rises in a layer-by-layer manner, allowing the following layer to be placed on the last. Scaffolds are programmed to support the structure while the thermoplastic extrudates harden together. These are either removed by solvents or machine tools. FDM 3D-printed products are considered not to be aesthetically pleasing due to the visible lines showing the layers. Acetone baths are used as a post-process to improve the finish. Hot-melt extrusion is a process which uses a polymer, such as acrylonitrile butadiene styrene (ABS) and polylactic acid (PLA), to create the filaments to be used in FDM [15].

### 2.5. Drop-On-Solid Deposition (DOS)

DOS is a form of powder-bed printing that consists of a bed containing powders which is sprayed with a liquid binder solution from an inkjet printhead. The thin layer of powder fuses together and another layer of powder is spread over the top of the existing bed. The unbound powder acts as a support during the process, preventing collapse. This process is repeated until the object is complete, where the excess powder is brushed away. Although this method is unable to create hollow objects, its main advantage is modifying excipients within the powder bed, allowing for different compositions and avoiding heat treatment for thermally unstable drugs [18]. When this technique is applied to the field of pharmaceutics, it allows for exact drug dosage to be created in a specific location.

## 3. 3DP in Neuroscience

### 3.1. Surgical Planning/Training

Neurosurgery is regarded as one of the most intricate medical specialties which require a high-quality procedural outcome. Although radiologic imaging techniques have advanced, creating 3D computer tomographic angiography and digital subtraction angiography, both imaging techniques are primarily for inspecting blood vessels and tissues to diagnose diseases, such as cerebral aneurysms. The images are still visualized on a 2D platform, allowing no physical interaction. Three-dimensional printing allows interaction with physical models which are created to a high-fidelity and real patient specificity.

Three-dimensional printing was initially introduced to the healthcare sector for craniomaxillofacial surgery [19]. Later, 3DP methods were used to reproduce cerebral vasculature morphology in 1999 [14]. Five years later, SLA printing technology was used to print precise models of the cerebral aneurysm [20]. This encouraged many neurosurgeons to use SLA. A few years later, vascular models were created by researchers but there were several issues such as being time-consuming (it took several days to print the required model) and lack of uniformity in the thickness of vessel walls, which negatively affected accuracies in clipping [21]. In 2015, Mashiko et al. accelerated the production of the models, where the required models were printed in several hours rather than several days; however, once again, these models suffered from discrepancies in vessel wall thickness, elasticity, and adhesion [22].

Three-dimensional printing has the ability to help in pre-operative planning, educating surgeons in training, and enhancing research in neurosurgery. With the help of 3D printing technology, it is possible to generate devices, such as tools and implants, that change how surgery is performed. Computed tomography angiography (CTA) and magnetic resonance imaging (MRI) images were converted into software, compatible with the stereolithography device [23]. Before printing, reconstructions were created by volume rendering techniques, image thresholding allowed segmentation between vessels and bones, and a 3D connectivity function (a software option) disconnected the main article tree from soft tissue in the brain. This modified data were then used to print the final model. The stereolithography device uses a UV-light beam to solidify a photosensitive liquid resin monomer layer by layer. Each layer is coherent with modified data. The polymer was then rigidified by curing in an ultraviolet oven. Post-operation, neurosurgeons evaluated the modeling, reporting the aneurysm relationship and cerebral vasculature as being precise. Models were able to reduce the time of comprehension of the aneurysm as it did not require mental reconstruction from several images. Within the report, it was noted that the most appropriate angle for surgery could be identified, therefore helping plan the patient’s head position prior to surgery. Having a greater understanding of the patient’s anatomy could also increase surgeons’ confidence in the procedure. The model also allowed surgeons to measure the correct aneurysm clip. This has the potential to reduce the time of surgery if it were conducted on the model before the operation, which poses real benefits to patients and has led to other studies.

Cerebral aneurysm surgery requires an experienced surgeon and complex microsurgical clipping, which is the use of a small metal clip to stop blood flow into the aneurysm, thus creating a need for effective training. Kimura et al. [21] created elastic hollow models of cerebral aneurysms to emulate this surgical procedure. Data from CTA images were transformed into a stereolithographic format and then adjusted to fit a rapid prototyping machine. A rubber-like polymer (FullCure 930 tango plus) was sprayed at 0.03 mm thick for each layer and then hardened by UV light; these layers pile up creating the vessel walls. Cranial-based bone was constructed using the same technique, barring the polymer which had talc and resin (becoming hardened when irradiated). Access to the aneurysm was simulated by craniotomy. When performing the pre-operation on the model through an operating microscope, multiple clips were applied to determine the correct angle, shape, and size. Surgeons were able to select the correct one prior to the real neurosurgery. Live surgery, however, does include haptic feedback during the procedure as well as biomechanical properties of the arteries, which are important but were not emulated in this model. Due to the constraints of the scanning data, the thickness of the vessel walls could not be obtained, so was estimated to be around 0.3–0.5 mm. Missing parameters affect the accuracy of the model. Overall, it may help trainee neurosurgeons to practice techniques and strategies as well as possibly reduce errors in surgery [21].

**Figure 1:**
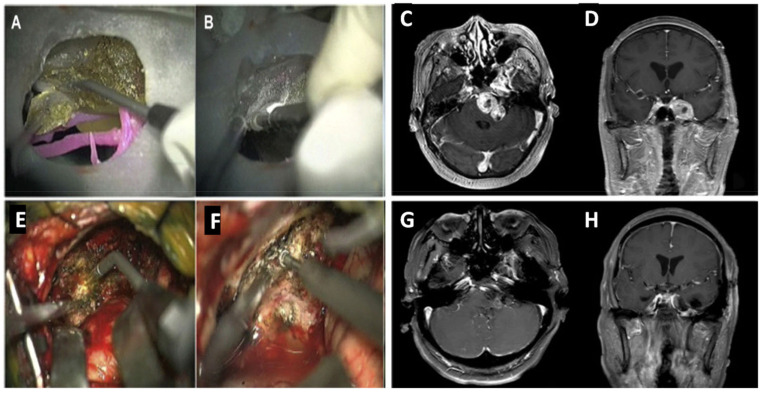
Simulated surgery and intraoperative scenario. (**A**)Three-dimensional tumor model resection using left subtemporal keyhole approach and laser-knife. (**B**) Surgical simulation of removing the residual tumor on the 3D model from the petrous apex after rotation. (**C**) MRI scan at transverse position displaying tumor growth through middle-posterior cranial fossa preoperation. (**D**) Tumor observed in the coronal position, preoperation. (**E**) Real-patient tumor removal with laser-knife. (**F**) Intraoperation grinding of the petrous apexin. (**G**) Postoperation MRI scan showing no residual tumor after surgery in transverse view. (**H**) Coronal position confirming no tumor remaining post operation [24].

A more recent study constructed models with skull base, cerebral arteries, and brain tumors or aneurysms for simulated surgery [24]. Similarly, the authors of the study used real patient data from computed tomography (CT) scans, MRI, and CTA to obtain imaging data about the aneurysm or tumor. A tectal longitudinal column (TLC) allowed digital information to be collected about the craniocerebral structure. In contrast to previous methods, the PolyJet printing method was used to print the models. Different photosensitive polymers were used, allowing for individual textures and colors, solid skulls, and flexible blood vessels. The first 3D-printed model for brain tumor dissection was conducted. After simulated surgery on the model of patient 1, all tumors appeared to be removed when viewed through a microscope at the middle cranial fossa and posterior fossa (Figure 1A). However, when the model was rotated, the residual tumor could be seen beneath the petrous bone (Figure 1B).

The model of patient 2 allowed surgeons to practice separating adhesions between the tumor and blood vessels as it had high spatial correspondence. However, limitations included the texture of the tumor and protection of intracranial nerves as well as hemodynamics. As 3D technology advances, different techniques and materials may allow for the addition of these. After simulated surgeries with models, real-life surgery took place, allowing surgeons to put their knowledge into practice. Figure 1G,H are MRI images confirming that no residual tumor remained in the patient after real-life surgery. Although images from simulated surgery (Figure 1A,B) are not identical to the real-life surgery (Figure 1E,F), it did not prevent refining the strategic planning for surgery. Overall, this study indicated that the experience gained from the simulated surgery prevented unnecessary exploration, producing a minimally invasive, faster, and more effective surgery, ultimately improving the outcome for patients [25].

Exposure to some procedures such as synostosis surgery is rare because the presence of craniosynostosis is uncommon. Therefore, it is challenging for trainees to gain practical experience during their training [25]. Three-dimensional-printed models provide a platform for trainees to practice uncommon cases. Deep-seated tumor biopsy is a complex procedure that requires a variety of tools and devices that trainees must learn to use, such as navigation system endoscopes, microscopes, and drills [25]. Both surgeries mentioned above can be replicated in 3D-printed models, allowing a platform for trainees to practice using equipment. Karuppiah et al. [25] created a training model of a pineal region tumor using 3D printing. Before practicing with the model, trainees received lectures on performing a frameless stereotactic biopsy. This involved planning, positioning of patient, equipment, and execution of the procedure. Trainees were then allowed to practice all the various steps on the model in one sitting without the pressure of making mistakes on a real patient. Inadequate steps or sub-optimally performed steps were repeated on the model to allow the trainees to improve their technique. In this example, 3D-printed models help surgeons reach competencies faster and improve their confidence without risk to patients [25]. Three-dimensional-printed models are likely to play a greater role in the future of neurosurgical training due to their educational benefits.

A review by Thiong’o et al. [26] collected international literature concerning 3D printing of brain and cranial vault pathology to define the global utilization of 3D printing within neurosurgical education. They found a general positive trend in relevant publications between 2014 and 2019. In addition, this review highlighted areas within neurosurgery that were more common in using phantom simulations such as neurovascular and skull-based techniques in comparison to resective epilepsy surgery and brain tumor microdissection techniques. This finding is possibly due to the fine dissection detail required for resective epilepsy surgery and brain tumor microdissection techniques and 3D printing techniques at the time not producing finely detailed models for simulated surgery [26]. As technology advances in the coming years, these uncommon areas for neurosurgical simulation could see development.

**Figure 2:**
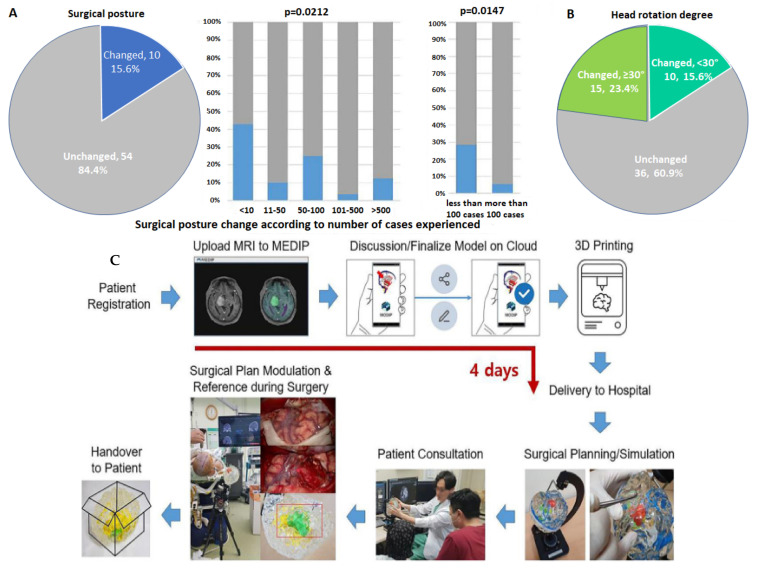
(**A**) Percentage of cases that changed surgical posture when looking at 3DP model of a brain tumor after MR images. The number of cases the surgeons treated before is shown against the percentage of change. (**B**) Percentage of cases that changed the degree of head rotation once presented with 3DP model of a brain tumor. (**C**) A flow diagram showing the implementation of 3DP model of a brain tumor in a clinical setting [27].

Another study, by Dho et al. [27], established a 3D-printed model production system, as seen in Figure 2C. The efficiency was tested in a clinical field by multiple neurosurgeons evaluating 64 cases in total, once from MR images and then again from 3DP brain tumor models. Figure 2A indicates the percentage of change in surgical planning after looking at the 3D models. Interestingly, surgeons who have experienced fewer than 10 surgical cases changed 42.9% of the decisions after viewing the 3D model.

Furthermore, a study by Ploch et al. [28] gave 10 neurosurgeons a multicenter surgical survey to give feedback on a 3DP personalized model of the brain made from soft gelatin. The 3DP models were fabricated within 24 h and cost USD 50 to produce. The participants in the survey perceived the model to be very useful for surgical planning and preoperative planning, with both scoring 95% usage satisfaction (US) in the survey [28]. The models also obtained US of 94.44% for teaching, 100% for learning, and 85% for patient illustration. Although the anatomy, visual appearance, and model size scored between 81% and 100% on model satisfaction, it was noted that further details such as the meninges and brainstem needed to be included within an enclosed skull [29]. Other parameters, such as intracranial pressure, could not be monitored in these models. The models’ satisfaction for stiffness, cutting properties, and haptic anatomy ranged from 43 to 57%, suggesting that the tactile experience of how the brain feels, moves, and responds to contact in surgery still needs to be improved upon in 3D models [28].

Overall, 3DP technologies allow for noninvasive and advantageous ways of visualizing anatomy that can be used in multiple areas in the medical field: diagnosis, surgical planning, and education. Current neurosurgical training methods use cadaveric heads which are a limited resource, require strict storage, lack pathological models, and are costly. Using 3DP models would increase the availability of training, thereby improving surgical outcomes due to more practice. Furthermore, 3D models could help with education from home, helping reduce contact with patients for trainees, which is becoming more relevant due to COVID-19 restrictions.

### 3.2. Patient Counseling

Education does not just apply to training young neurosurgeons. During patient counseling, 3DP models have been used to enhance patient comprehension. Craniosynostosis patients had specific 3DP models of their skull printed, which were used to help neurosurgeons explain the craniofacial deformity to patients’ parents, allowing them to evaluate consent on surgical decisions [29]. Questionnaires were provided to and answered by the parents: once, after an explanation with CT images, and again, after it was repeated with 3DP models. Results indicated that participants had a clear understanding of 3DP models and there was no need to study the condition further. Surgical management preferred 3DP models to CT images (*p* = 0.028) as they assisted with surgical decision-making [29]. Models were affordable at a mean cost of USD 5.20 [29] This study reveals how 3DP technology could aid clinical practice. Another study on the utilization of 3D printing in pediatric neurosurgery found that 3D-printed models aided family members of the patients’ understanding of the surgery as well as non-surgical members involved in the procedure [25].

### 3.3. Implants

**Figure 3:**
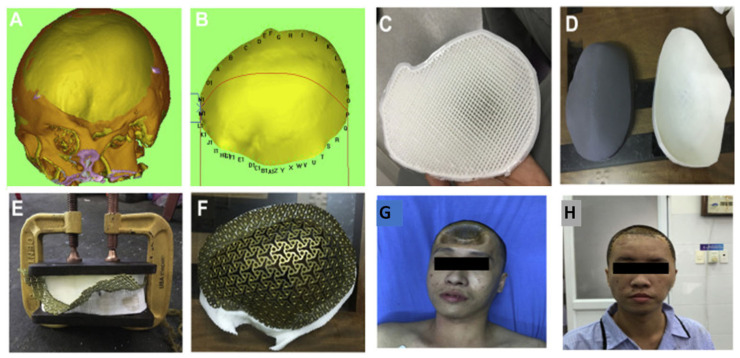
(**A**) ROBINS 3D image-reading software of patients’ skulls with defects. (**B**) Isolated cranial defect. (**C**) Polyurethan model used to make (**D**) cement moulds. (**E**) Moulds clamped together with titanium mesh in between. (**F**) Completed prosthetic with a specific contour for skull defect. (**G**) Patient’s head before cranioplasty indicating cranial defects in frontal regions. (**H**) Patient’s head after customized titanium implant, presenting improved aesthetic outcome due to restoration of calvarium contour [12].

Advancements in 3DP allowed for the production of customized bone prosthetics. Cranioplasty is an operation to repair a bone defect in the skull. With the help of 3DP, synthetic implants can be custom-made and anatomically precise to fill even the most complex areas, for example, the pterion and frontal region. Using thermoplastic polyurethane, an FDM printer makes an implant that adheres to cranial defects from ROBINS 3D imaging rendering software (Figure 3A,B). The polyurethane model is used to make cement moulds. In between these two moulds, a titanium mesh is placed and clamped; this pressure contours the mesh to the skull defect (Figure 3E). Final preparations include trimming excess titanium and sterilization, making the manufacturing process last 5 days overall [7]. All implants had “excellent anatomic fit” and did not need removal or revision after 6 months. Only 14.3% of these implants had complications during surgery. All patients had improved aesthetic outcomes (example shown in Figure 3G,H before and after implant) and were satisfied with the restoration. This new technique was created to allow lower-to-middle income countries to increase patient care with a low-cost and self-sustainable method. Three-dimensional printing may help to allow greater sustainable access to healthcare across the world.

There is also a need for patient-specific spinal implants. Surgeons used 3D virtual planning and 3D-printed models to reduce the curvature of the spine in the case of kyphoscoliosis [30]. What was unique to this case was the 3D-printed personalized osteotomy-guiding templates that helped plan pedicle subtraction osteotomy (PSO) in surgery. These templates were made by the SLS process from polyamide. Three-dimensional templates were only useful in the first stages of PSO as rods needing to be placed for stabilization. The implementation of rod inlets in future 3D-printed designs has been discussed. Furthermore, it is speculated that it could contribute to a safer procedure; however, there were no qualitative data showing that using these templates would improve patients’ outcomes.

### 3.4. Customized Instruments

Instruments used in the medical field have been 3D printed. Magnetoencephalography allows brain activity to be recorded, although it is limited by co-registration to anatomy or head movement. These errors can be minimized by using SLS methods to print subject-specific head-casts [31]. Another example is a proton range compensator (RC), which normally uses a computerized milling machine (CMM), requiring a water-purification and noise-suppression system needing large facilities. The RC protects organs near tumor tissue during proton therapy by providing a conformal dose distribution. An RC was successfully 3D printed using acrylic plastic cured by UV and showed no significant difference in dosimetric characteristics compared with an RC made by CMM [5]. The 3D printing time was 15 h longer than CMM, but the system requirements were minimized [5].

**Figure 4:**
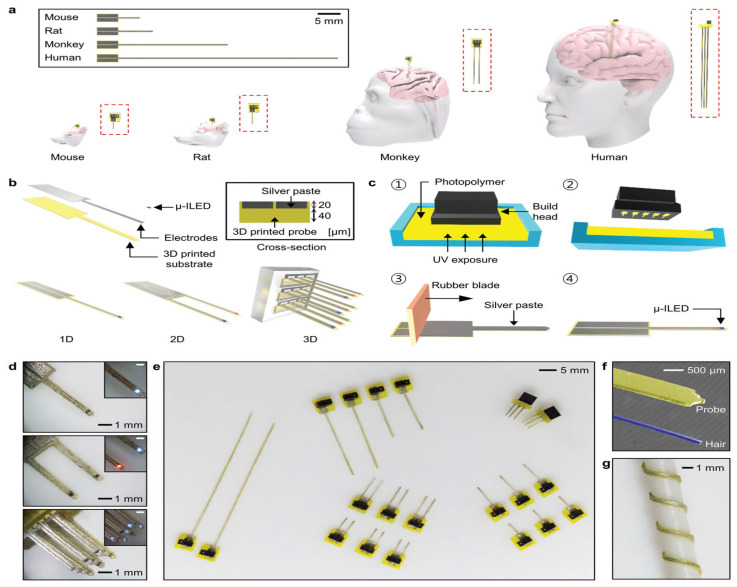
(**a**) Image demonstrates the customizability in terms of scale, allowing for various mammalian brains to use 3D-printed optogenetic probes (3DP-OPs). (**b**) Design and architecture of 3DP-OPs. (**c**) Manufacturing sets of 3DP-Ops: (①) printing probe via SLA, (②) removal of probe and washing off excess residue, (③) application of silver paste to microgroove surface of probe and transverse the surface longitudinally with a rubber blade, (④) attaching microscale inorganic light emitting diode (µ-ILED) to probe tip. (**d**) Optical images indicating customizability in structural design and different colored µ-ILEDs. (**e**) Printed 3DP-OPs showing different lengths (**f**) Thickness of the 3DP-OPs (yellow) against that of a human hair (blue). (**g**) Flexibility of the 3DP-OPs, which are wrapped around a 1.5 mm rod [6].

3D printing is not limited to medical applications; it can also be a part of discovery and research in the field of neuroscience. Techniques related to neuroscience often use highly specific tools which could be 3D printed. Optical neural probes used in optogenetics (a selective neuromodulation technique which uses light to manipulate neural circuits) currently rely on expensive and inconvenient cleanroom facilities in order to be manufactured. A 2020 study presented a new way of manufacturing optical neural probes by using SLA [6]. The SLA device provides a low-cost mass production and customizable head (shown in Figure 4c①,c②) enabling 50 distance probes to be printed at once. The CAD process allows an on-the-fly design optimizing measurements for a variety of animal models/brain structures (illustrated in Figure 4a). Post-processes include: washing to remove excess residue, a rubber blade that smooths a silver paste into the microgrooves of the electrode, soldering of μ-ILED into the tip, and a coat of polydimethylsiloxane bilayer ensuring biocompatibility. In comparison to fiber-optic probes (traditional probes), the 3D-printed probe indicated longer longevity in in vivo experiments and exerted smaller amounts of mechanical stress when implanted. Overall, these studies demonstrated the ability of 3D printing to make advanced tools in medical/theoretical neuroscience as well as decreasing their cost.

### 3.5. Nasal Cast in Nose-to-Brain Delivery Development

Instrument nose-to-brain delivery presents many benefits when targeting the central nervous system (CNS) with drugs: short latency, bypassing the blood–brain barrier, increased bioavailability in CNS, noninvasive, fewer side effects, and avoidance of systemic toxicology [32]. It is an interesting route of drug delivery for severe brain pathology (i.e., brain tumor) and neural degenerative diseases (Alzheimer’s and Parkinson’s diseases). In order for the drug to reach the brain, it must reach the olfactory zone at the top of the nasal cavities in which it needs to diffuse through the nasal mucosa and the olfactory nerves toward the brain [33,34,35]. This route of drug delivery requires a significant fraction of the dose to be deposited into the olfactory zone in a consistent manner, which requires expert knowledge in formulation and an understanding of the complex geometry of cavities. Three-dimensional-printed nasal cavities, also known as nasal casts, are a valuable tool to bridge and test the formulation, nasal administration device, and the drug journey to the nasal cavities to give crucial information about the efficiency of a nasal drug product [36].

One of the first barriers to this delivery method is the nasal vestibule and nasal valve, which prevent the drug from reaching the olfactory bulb. Three-dimensional-printed nasal casts have been used to determine the ideal conditions to deposit the drug on the olfactory bulb. All four methods mentioned above have been used to create nasal cases, and out of them, SLA, SLS, and PolyJet all produced similar deposition profiles. Because FDM uses thermoplastic filaments, it experienced thermal shrinkage which was problematic for the precision of the casts. Casts also became porous due to delamination, and the plastics used in this method reacted with solvents used for drug discovery, ultimately making FDM a poor process for making nasal cases [36]. Colorimeter gels were used to quantify the distribution of liquid spray. The material used to make the cast, therefore, had to be transparent to obtain the color intensity [37]. Casts are usually produced by milling, which is precise despite needing skilled staff and expensive equipment. On the other hand, 3DP does not require either while retaining the same level of accuracy, making it a worthy replacement. In addition, CT scans allowed for patient-specific casts to be printed, permitting the optimization of drugs entering the brain by personalized administration. Experiments with these casts could not only improve drug deposition but also lead to a more effective design of inhalation devices [36]. However, it should be noted that the nasal cast does not provide some crucial information such as drug diffusion through the nasal mucosa and drug bioavailability in the brain. Overall, the study suggested that a second clinical trial with 3DP nasal casts was needed to validate the test process as well as in vivo testing on mice as it presented a more realistic model.

## 4. 3D Bioprinting

Three-dimensional bioprinting is similar to 3DP; however, it uses biomaterial/viable cells instead of polymers to print engineered tissue. It is a developing field in regenerative medicine as it allows for patient-specific spatial geometry, modulated microstructure, and fabrication of tissue scaffolds from the precise placement of individual cell types [38]. A lowered rejection risk, uniform tissue growth in vivo, and integration with host tissue are just a few of the benefits homogeneous hydrogel scaffolds produce [39].

First, a virtual model is created of the defect with imaging modalities such as CT/MRI. Next, CAD is used to design the architecture of the scaffold. This design often includes biomimicry of the selected tissue. This is followed by a selection of cell types, materials, and bioactive molecules to prepare the bioink. Viscosity, gelation, and crosslinking capabilities need to be considered in this selection process as the hydrogel properties need to provide a balance between cell suspension and structural fidelity. The scaffold is then printed using one of many bioprinting techniques (inkjet-based, extrusion-based, laser-assisted) and is then either incubated or directly transplanted [39]. Throughout the process, it is important that scaffolds are provided with oxygen/nutrients as well as an efflux of CO_2_/by-products to avoid necrosis. Therefore, vascularization or porosity is incorporated into scaffolds [40].

Currently, autologous nerve grafting is used for peripheral nerve injury despite its many limitations, such as loss of donor function, limited donors, and misalignment [41]. Bioprinting nerve grafts could mimic the mechanical structural and cellular properties of peripheral nervous system tissue while removing these constraints. Schwann cells were successfully bioprinted using alginate-hydrogels for peripheral nerve regeneration and produced an expression of neurotrophic factors [42]. This method used rat Schwann cells which were cultured at 37 °C in a humidified atmosphere. Four days later, they were resuspended in a culture medium after being digested by trypsin and then mixed with hydrogel precursor. The result was a solution with concentrations of 8% gelatin, 2% sodium alginate, 2 × 10^6^ cells/mL. This aqueous gel was kept in a syringe at 28 °C and extruded onto a plate with a room temperature of 20 °C, allowing gelatin-alginate to be liquid until deposition, where it becomes gel rapidly. The printed scaffolds were immersed for 5 min in CaCl_2_ at 50 mM for crosslinking. Unfortunately, when these were transplanted into rats in vivo, the scaffolds were susceptible to changes in volume, degradation, and morphology. Further research could be conducted on developing a material with greater strength. Another restriction was the nozzle diameter (160 μm) as it was greater than an axon, preventing the optimal resolution of scaffolds [42]. Although the transplantation was unsuccessful, the in vitro cells were successful. This bioprinted material could be used in toxicology testing and pathophysiology of disease [43], reducing animal testing, and could prove more reliable and effective if they were human-derived.

Another study used human neural stem cells (hNSCs) to construct neural tissue [44]. Frontal cortical hNSCs in conjunction with bioink comprising polysaccharides alginate, carboxymethyl-chitosan, and agarose formed chemical crosslinks following microextrusion bioprinting. Carboxymethyl-chitosan aids cell survival within the scaffold. Alginate allows for the presence of cations while gelation occurs and agarose permits correct bioink viscosity, both contributing to the constructs’ structural support. Bioprinting was followed by in situ differentiation which principally resulted in GABAergic neurons, glial cells, and self-renewing cells that continued to proliferate for 10 days. The neural mini-tissue constructs (nMTCs) produced indicated spontaneous activity [44]. This experiment demonstrates a potential platform to study human neurodevelopment and disease as well as patient-specific drug screening in vitro. However, results are biased toward GABAergic neurons, and more research should be conducted into adapting this method to create a glutamatergic/serotonergic agent to allow for different modeling systems.

## 5. 3DP Drugs, Polypill, and Customizable Design

Currently, there is only one 3DP drug on the market, which is Spritam for epilepsy patients and which was approved by the FDA in 2015. It uses ZipDose technology, which is a form of DOS. The drug has a soluble matrix with porosity allowing it to dissolve quickly in water, making it more efficient than conventional tablets [45]. It is also available in spearmint flavor and able to be manufactured on a large scale. More recently, studies using ultraviolet curing have made ropinirole hydrochloride tablets, a dopamine agonist used in the treatment of Parkinson’s [46]. Photoinitiators initiate crosslinking by generating free radicals/cations/anions when Spritam is illuminated by UV light. This is similar to photosensitive liquid resin in SLA. Tablets released 60% of active pharmaceutical ingredients (APIs) after the first hour, increasing to 89% after 4 h. Although drugs can be 3D printed, the technology presents some disadvantages in comparison to current manufacturing methods, for example, the production yield per given time is substantially lower than conventional methods. On the other hand, the 3DP drug allows for customizations in design, and multiple medications in one tablet which could potentially have personalized dosage and absorption for the individual. Furthermore, 3DP drug allows for many parameters which can affect the drug release rate such as infill percentages, shape/geometry, and thermal processing parameters on the printed formulations [17].

The idea of a polypill represents one tablet that involves a combination of multiple drugs. This could be extremely useful for patients suffering from dementia who forget their medication as well as for caregivers [47]. Remembering one pill in the morning as opposed to ten throughout the day at different times is more achievable, thereby reducing the risk of mistakes [48]. Polypharmacy is the regular use of five or more medications for one patient, which is typically common in geriatric patients. Multiple prescriptions can increase the risk of adverse medical outcomes [49]. Multiple medications can cause potential interactions between drugs, which can lead to complications for patients. Additionally, multiple tablets can lead to a decrease in medication adherence, with dosing times/intervals as well as administration being forgotten [50]. Due to this, one tablet with several active pharmaceutical ingredients (APIs) is ideal for pharmaceutical companies to improve patient adherence. In contrast, allergic reactions or side effects might make it harder to differentiate between which API is causing the reaction. For these reasons, it could be suggested that poly 3DP drugs are not ideal for patients who are ingesting an API for the first time and are more suited to patients with a regimen already in place. Although 3D-printed drugs are easy to titrate due to the printing being highly customizable, this may not be the case if the 3D printing devices are not widely available.

**Figure 5:**
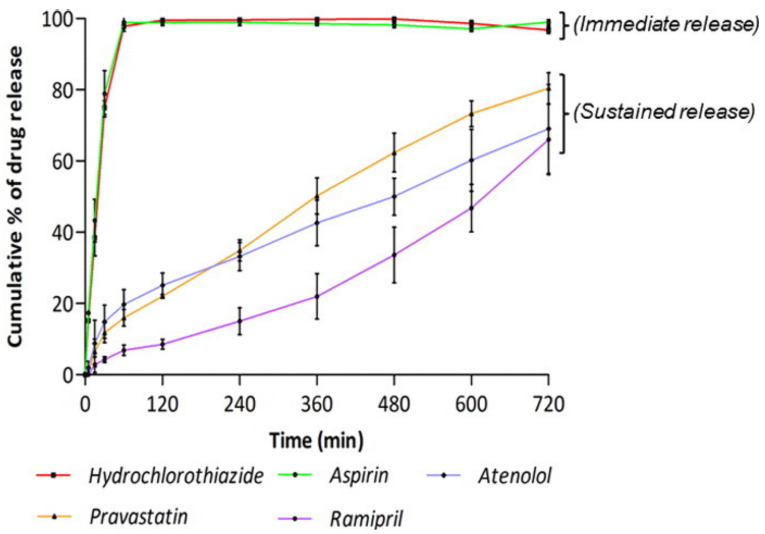
The drug-release profile of each drug from the compartments in the polypill in vitro [50].

Fused deposition modeling 3D printers have been widely applied in 3D drug printing; however, the main issue is the possible heat-induced degradation of thermally sensitive drugs. Different drugs within the polypill will have different levels of tolerance to heat. A study conducted in 2015 by Khaled et al. [51] employed a room-temperature extrusion system to produce a polypill. First, a hydrophobic cellulose acetate shell was extruded. Then, a hydrophilic matrix of the active drugs (atenolol, pravastatin, and ramipril) was extruded into a sustained-release compartment of the acetate shell. A disintegrant, sodium starch glycolate, was used to combine aspirin and hydrochlorothiazide, which was extruded into the immediate-release compartment. This compartment was on top of the sustained-release compartment [51]. This technique allowed for customization; raised dots on the top surface allow for visual and touch identification, perfect for the visually impaired. The polypills were also mechanically stable and could be handled without structural integrity being lost. As seen in Figure 5, this experiment was able to create different drug-release profiles in vitro from one tablet. After one hour, almost 100% of hydrochlorothiazide and aspirin were released due to the sodium starch glycolate, which rapidly absorbs water. In contrast, the drugs contained in the sustained-release section required 720 min to release around 66% of APIs due to the gel-like formation of the hydrophilic matrix [51].

Although these drugs are not directly related to neurological patients, the concept of a polypill with different release rates could greatly impact this group. Polypills should be tested using the SLA ultraviolet curing techniques mentioned above to avoid thermal degradation. By using the SLA technology, a tailored polypills with six different APIs was able to be fabricated, providing evidence that 3DP polypills might be a possibility in the near future [52].

Disinhibition of cholinergic neurons and increased glutamatergic activity is a result of the progressive degeneration of dopaminergic neurons in the substantia nigra [53]. This imbalance in transmission creates symptoms such as tremors, akinesia, rigor, and bradyphrenia in Parkinson’s disease (PD), which is usually treated with polytherapy. Although levodopa (LD) and benserazide (BZ) or carbidopa (CD) are effective ways to relieve symptoms for PD patients [54], many experience motor fluctuation when undergoing LD therapy and sometimes experience LD-induced dyskinesias [55]. The causality behind these fluctuations is poorly understood; however, there is a strong correction between dyskinesias and dose-by-dose LD plasma pharmacokinetics [56]. When LD plasma levels exceed the threshold of improvement for bradykinesia, LD-induced dyskinesias frequently occurs; hence, there is a need for optimal management for LD therapies in PD. The Accordion Pill is a novel delivery system with polymetric films which contains two APIs: immediate-release CD; immediate and controlled-release LD [57]. This pill is designed to extend gastrointestinal release with the aim of a more stable LD plasma concentration within patients. A phase 2, two-way randomized crossover study with four cohorts of participants with PD compared both the Accordion Pill and immediate-release CD and LD. Results from this study showed more stable LD plasma concentration in PD patients and a significantly decreased C_max_. Furthermore, in comparison to immediate-release LD, the Accordion Pill significantly improved standard measures of motor symptoms. Further research could be conducted with 3D-printing technologies (e.g., FDM) to create a similar pill to the Accordion Pill as this manufacturing method can also create layers with different release profiles with multiple APIs.

Windoft et al. [58] developed an oral dosage form with pramipexole (PDM), LD, and BZ with FDM 3D-printing process, aimed at patients with PD. Hot-melt extrusion created all the filaments with 1.85 mm diameter for the FDM printing. The FDM printing process uses CAD, which allows for a variety of geometry in the design [59]. Different geometries can impact the surface area-to-volume ratio. In this case, hollow mini cylinders were printed which were thought to have been easier to swallow for patients with PD [58]. Due to the low density of the formulation, the tablet had a floating property which resulted in prolonged gastric residence time. This prolonged gastric residence time is crucial to saturate the transporter in the upper small intestine with LD over long periods of time. The long release time also increases the intake intervals, which can improve adherence.

## *6.* Limitations and Future Perspectives

There are many different types of 3D printers, and each one has its own advantages and disadvantages. These include the length of printing an object, the type of materials used, accuracy and resolution, and durability. Although serious improvements in 3D technology have been made, there is no standard printing process for patient care and medical research. In order for neurosurgeons to obtain the benefits of 3D printing technology, researchers involved in the production of 3D printers need to optimize the use of additive manufacturing technologies and find a way to overcome the limitations if 3D printer use is to become common in the neurosurgical field. For 3D printer manufacturers to make 3D printers more appealing to neurosurgeons for the treatment of neurological diseases, comprehensive improvements are essential in 3D printing hardware and CAD software. Some of the limitations of the application of 3D printers in neurological diseases could be the speed of the 3D printing, cost, resolution, and texture. Although several 3D printers such as Makerbot Replicator, Objet260 Connex3, and ProJet 6000 have been employed in neurosurgery, they are still unable to produce realistic models (e.g., texture and hemodynamic factors). With the advancement and high transferability of 3D printing technology, it could likely find its place in clinical use provided that the major limitations of this technology in neurological diseases are overcome.

## 7. Conclusions

Three-dimensional technology is branching into many fields in neuroscience including but not limited to medical, education, research, bioengineering, and pharmaceutics. This manufacturing method is expected to be used more frequently due to the decrease in cost, ease of use, and advancements in technology. Planning and presurgical simulation can be enhanced by patient-specific 3D models while improving training for young neurosurgeons. Communication with patients is also more efficient with 3D models. Three-dimensional-printed implants and tools can be used in medical practice and research with increasing accuracy. This will lead to advancements in neuroscience techniques and research methods, in time increasing understanding of the nervous system and pathologies, allowing for improvements in treatment. Bioprinting may provide new ways of testing drugs and a possible new method for regenerating nerves. Three-dimensional drug printing is helping to personalize medicine, making it more effective and easier to use for the individual. Overall, it can be expected that 3D technology will, consequently, indirectly improve the effectiveness of treatments and outcomes for patients with neurological diseases.

## Data Availability

Not applicable.

## Abbreviations

3DP	Three-dimensional printing
NHS	UK National Health Service
CAD	Computer-aided design
SLA	Stereolithography
SLS	Selective laser sintering
FDM	Fused deposition modeling
DOS	Drop-on solid deposition
ABS	Acrylonitrile butadiene styrene
PLA	Polylactic acid
CTA	Computed tomography angiography
MRI	Magnetic resonance imaging
CT	Computed tomography
TLC	Tectal longitudinal column
PSO	Pedicle subtraction osteotomy
RC	Range compensator
CMM	Computerized milling machine
3DP-OPs	Three-dimensional printed optogenetic probes
CNS	Central nervous system
hNSCs	Human neural stem cells
nMTC	Neural minitissue constructs
APIs	Active pharmaceutical ingredient
PD	Parkinson’s disease
LD	Levodopa
BZ	Benserazide
CD	Carbidopa
